# Study of Antibodies to Influenza Neuraminidase N2

**DOI:** 10.3390/ph15050498

**Published:** 2022-04-19

**Authors:** Yulia Desheva, Nadezhda Petkova, Tatiana Smolonogina, Svetlana Donina, Alexey Go

**Affiliations:** 1 Scientific and Educational Center Molecular Bases of Interaction of Microorganisms and Human of the World-Class Research Center Center for Personalized Medicine of Federal State Budgetary Scientific Institution “Institute of Experimental Medicine”, Saint Petersburg 197376, Russia; pn.nadezhda@yandex.ru (N.P.); smolonogina@mail.ru (T.S.); sveta.donina@gmail.com (S.D.); 2 Medical Center, St. Petersburg Research Institute of Epidemiology and Microbiology Named after Pasteur, Saint Petersburg 197101, Russia; alexeigo@mail.ru

**Keywords:** influenza, neuraminidase, antibodies, live influenza vaccine

## Abstract

Humoral immunity to influenza neuraminidase (NA) was evaluated among different groups of people including patients with acute influenza infection and healthy people in different age groups using an enzyme linked lectin assay (ELLA). The amino acid composition of NA of seasonal influenza viruses A/Victoria/361/2011(H3N2) and A/Hong Kong/4801/2014(H3N2) differed by 2%, while cross-reacting neuraminidase-inhibiting (NI) antibodies to them in the same serum samples were detected in 10% of cases. Middle-aged patients born from 1977 to 2000 had a high level of hemagglutination-inhibiting (HI) antibodies to A/Hong Kong/4801/2014(H3N2), but almost no NI antibodies, which may indicate that in the case of a change in the hemagglutinin (HA) subtype, this age group will be susceptible to influenza A/H3N2 viruses. Therefore, it could mean there is a need for priority vaccination of this age group with a vaccine against the appropriate strain. It was shown that after intranasal administration of live influenza vaccine (LAIV) for the 2017–2018 season, serum antibody response was not lower compared to that during natural infection. In older people, antibodies to archival A/H2N2 viruses were detected more often than to modern A/H3N2. Since the conversion of antibodies to HA and NA often did not coincide, antibodies to NA can serve as an additional criterion for assessing the immunogenicity of influenza vaccines.

## 1. Introduction

The influenza viruses cause annually about 3 to 5 million cases of severe illness, and about 290,000 to 650,000 influenza-related deaths [1]. Due to widespread complications and their severity, influenza poses a significant threat to public health. Influenza A and B viruses circulate among humans, causing seasonal epidemics and pandemics. Because of the high variability, variant influenza viruses appear each year. The main antigens of the influenza virus are surface glycoproteins –hemagglutinin (HA) and neuraminidase (NA). A total of 18 HA subtypes and 11 NA subtypes have been characterized [2]. Upon entering the human respiratory tract, the influenza virus attaches to the mucosa, while HA binds to receptors containing sialic acid. NA enzymatically cleaves mucosal sialic acids and thereby makes it easier for the virus to reach the epithelium of the respiratory tract [3]. Similarly, NA releases newly formed virions that are associated with the host cell and with each other [4].

HA is the dominant antigenic component of the influenza virus, and antibodies directed against HA serve as the main criterion for assessing the post-infection or post-vaccination humoral immune response to influenza. Anti-NA antibodies reduce the severity of the infection and may provide protection [5,6]. When creating vaccines against influenza viruses with a new HA subtype, researchers discovered that when assessed by the formation of HI antibodies, immunogenicity often did not correspond to the protective efficacy of the vaccine [7]. Therefore, it is important to study the putative contribution of antibodies against NA to the heterologous protection against viruses with a new HA subtype. There are few studies of the cross-protection of anti-NA antibodies, and they were carried out mainly for the NA subtype N1 of the pandemic H1N1 strain [8,9,10,11]. In 2009, a new influenza A/H1N1 pandemic was caused by a virus that included a unique combination of gene segments not previously seen in any of the viral isolates. Genetic sequence analysis showed that this pandemic virus resulted from several reassortments of avian, swine, and human viruses and likely circulated among pigs in North America for 10 years [12]. In this regard, there is a clear need to study the state of herd immunity to pandemic influenza viruses in high-risk patients in order to identify the most vulnerable target groups for priority vaccination. At the same time, one should not forget about another strain, the influenza A virus-H3N2, which may be more virulent than H1N1 [13].

Moreover, the detection of antibodies to NA may be used to provide data on population immunity in cases when new antigenic variants of influenza viruses emerge, since subjects often do not have antibodies to the modified HA. For example, there are no HI antibodies to viruses A/H5N1 in the population, but there are cross-reactive antibodies to NA [8]. The evaluation of anti-NA antibodies can also be used to study the immunogenicity of influenza vaccines. To date, the most specific method for detecting anti-NA antibodies is the enzyme-linked lectin assay (ELLA) which utilizes high-molecular substrates—glycoproteins containing neuraminic acid. ELLA directly measures the yield of the enzymatic reaction product in the presence/absence of a blood serum sample. The use of a high molecular weight substrate, such as fetuin, reveals the steric effects of shielding the catalytic site by antibodies bound to the antigen, while low molecular weight substrates can reach the enzymatic pocket even in the presence of inhibitory NA antibodies.

## 2. Results

A phylogenetic analysis of NA subtype N2 of human influenza vaccine strains isolated in different years, in particular strains A/17/Hong Kong/4801/2014 (H3N2) and A/Victoria/361/2011(H3N2), was carried out.

**Figure 1:**
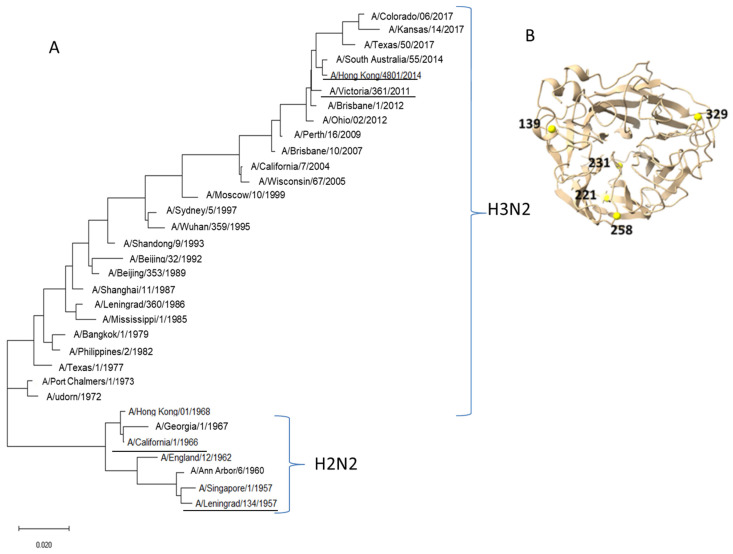
Phylogenetic analysis of NA of A/H2N2 and A/H3N2 influenza viruses. (**A**). The evolutionary history was inferred using the Neighbor-Joining method [14]. The optimal tree with the sum of branch length = 0.39463486 is shown. The tree is drawn to scale, with branch lengths in the same units as those of the evolutionary distances used to infer the phylogenetic tree. The evolutionary distances were computed using the Poisson correction method [15] and are in the units of the number of amino acid substitutions per site. This analysis involved 33 amino acid sequences. All ambiguous positions were removed for each sequence pair (pairwise deletion option). There was a total of 469 positions in the final dataset. (**B**). Spatial model of the globular part of NA (AA 83-469). Amino acids that are different in strains A/Hong Kong/4801/2014(H3N2) and A/Victoria/361/2011(H3N2) were noted.

The results of the phylogenetic analysis of NA amino-acid sequences of A/H2N2 and A/H3N2 influenza viruses are shown in Figure 1A.

The NA of the influenza virus A/Victoria/361/2011(H3N2) had a high degree of amino acid sequence homology (96–98%) with other A/H3N2 strains isolated from the human population from 2004 to 2018, as well as strain A/Hong Kong/4801/2014(H3N2). The most distant strains were isolated before 1968 (the degree of homology was 83–85%). The strains A/Victoria/361/2011(H3N2) and A/Hong Kong/4801/2014(H3N2) were 98% identical in amino acid composition. The strains differ by only 5 amino acid substitutions: E221D (Glu-Asp), V231I (Val-Ile), K258E (Lys-Glu), T329N (Thr-Asn), I392T (Ile-Thr). Amino acid substitutions were located in the NA “head” region outside the active sites. Using the Chimera software [16], a spatial model of the globular part of NA (AA 83-469) was created. In Figure 1B, the amino acids that are different in the A/Hong Kong/4801/2014(H3N2) and A/Victoria/361/2011(H3N2) strains are shown.

**Figure 2:**
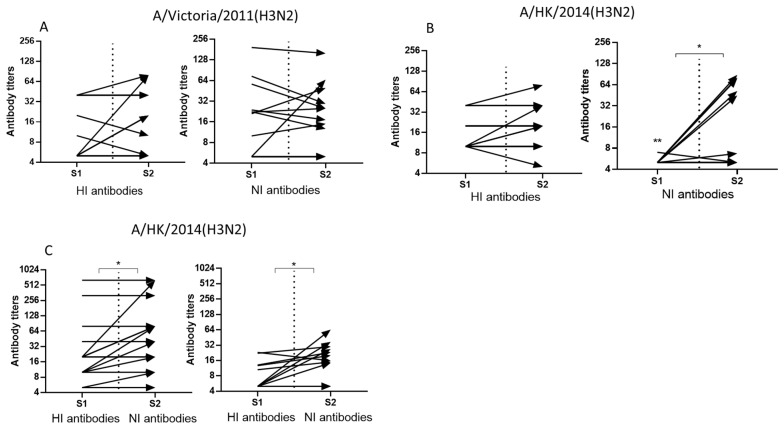
Antibodies to HA and NA of A/H3N2 influenza viruses in paired sera of patients with PCR confirmed influenza A infection. S1-sera obtained upon admission to the hospital, S1-sera obtained on days three to eight of hospital stay. (**A**)—antibodies to HA and NA of A/Victoria/361/2011(H3N2) in the sera obtained in 2017–2018 flu season (*n* = 15), * *p* < 0.05. (**B**)—antibodies to HA and NA of A/Hong Kong/4801/2014(H3N2) in the sera obtained in 2017–2018 flu season (*n* = 15), * *p* < 0.05; ** antibody levels at the start of hospitalization to A/Victoria/361/2011(H3N2) virus were significantly higher than to A/Hong Kong/4801/2014(H3N2) virus, *p* < 0.01. (**C**)—antibodies to HA and NA of A/Hong Kong/4801/2014(H3N2) in the sera obtained in 2018–2019 flu season (*n* = 20), * *p* < 0.05.

We assessed antibody conversions in pared sera of the patients with PCR confirmed influenza A infection. First, we evaluated the conversion of antibodies to the surface antigens of the A/Victoria/361/2011(H3N2) and A/Hong Kong/4801/2014(H3N2) influenza viruses in paired sera of patients hospitalized in the 2017–2018 flu season. In the 2017–2018 flu season, the mean levels of HI antibodies to both A/H3N2 viruses at the beginning of hospitalization were similar (*p* > 0.05), but the levels of NI antibodies were significantly higher to A/Victoria/361/2011(H3N2) than to A/Hong Kong/4801/2014(H3N2) virus (Figure 2A,B). The increase in mean antibody titers in the second blood sample was statistically significant only for antibodies to NA of A/Hong Kong/4801/2014(H3N2) virus (Figure 2B). This may indicate antigenic divergence between the NA of these two viruses, despite minor genetic differences.

Next, we assessed the formation of antibodies to HA and NA of A/Hong Kong/4801/2014(H3N2) virus in patients hospitalized with influenza in the 2018–2019 influenza season. Figure 2C shows that in these serum samples the average level of antibodies to the A/Hong Kong/4801/2014(H3N2) virus HA at the first point of the study was the same as in 2017–2018, but the level of antibodies to NA slightly increased. Despite the fact that the differences were not statistically significant, none of the patients in 2017–2018 had NI antibody levels exceeding 1:10, while in the 2018–2019 season five patients out of 20 examined already had NI antibody levels ≥1:10 and two ≥1:20.

Data in Table 1 suggest that seroconversions of antibodies to HA and NA of A/H3N2 influenza viruses occurred in 13–33% cases and GMT was 32–64 among those who responded with HI seroconversions, and 28–67 with seroconversions of NI antibodies.

**Figure 3:**
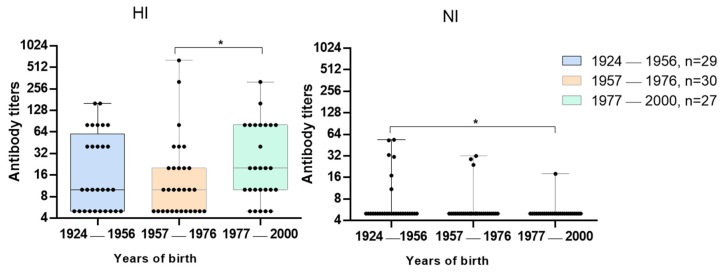
Serum antibody titers to the HA and NA of A/Hong Kong/4801/2014(H3N2) strain in healthy persons in 2017–2018 flu season, * *p* < 0.05.

The levels of antibodies to surface antigens of the A/Hong Kong/4801/2014(H3N2) strain were assessed in the sera collected from healthy persons of different ages examined in 2017–2018 flu season. For this, we used HI test with the A/17/Hong Kong/2014/8296(H3N2) vaccine strain and ELLA with A/H6N2 virus. The highest average level of HI antibodies was found among younger patients and the lowest among middle-aged patients (Figure 3).

The highest mean anti-NA antibody titers were found in the group of volunteers born between 1924 and 1956, while the lowest mean antibody titers were noted in the group of patients born between 1977 and 2000 (Figure 3).

‘Herd’ immunity was assessed by the proportion of individuals with protective HI antibody titers ≥1:40 [11]. For NI antibodies, protective titers have not been precisely established. Considering that the titers of antibodies to A/Hong Kong/4801/2014(H3N2) NI were generally low, we used NI antibody titers of 1:20 in this particular case (Table 2). Among the oldest and youngest patients there were similar proportions of individuals with HI antibody titers to A/Hong Kong/4801/2014(H3N2) (41.3% and 37%, respectively), and the geometric mean titers (GMT) of HI antibodies among the younger group were 22.7, and among the older they were 17.3. This may indicate that these age groups met with the A/Hong Kong/4801/2014(H3N2) influenza virus with the same frequency. However, among the youngest patients, NI antibody titers ≥1:20 were not detected (Table 2).

The lowest level of protective HI antibody titers to A/Hong Kong/4801/2014(H3N2) virus was in the group of middle-aged patients, while 10% of them had NI antibody titers ≥1:20.

**Figure 4:**
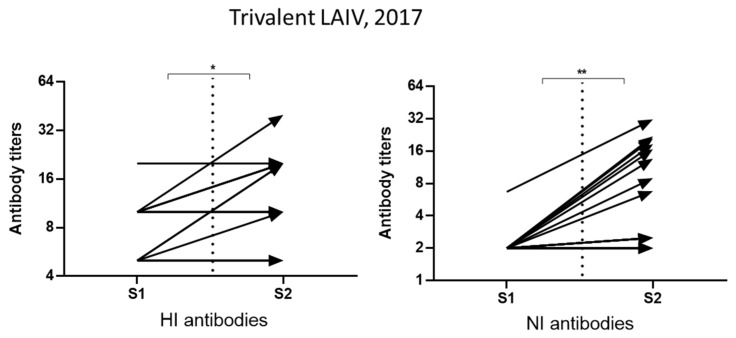
The results of the study of the immunogenicity of the A/H3N2 component of the trivalent LAIV (A/Hong Kong/4801/2014(H3N2)) during the 2017–2018 epidemic season, *n* = 18. A/17/Victoria/2011/89 (H3N2) virus was used for HI antibody detection, A/H6N2 virus was used for ELLA. S1–sera collected before vaccination, S2–sera collected three weeks after vaccination. * *p* < 0.05, ** *p* < 0.01.

Antibodies to NA of A/Hong Kong/4801/2014(H3N2) influenza virus were also evaluated in individuals vaccinated with trivalent live influenza vaccine (LAIV) in the fall of 2017. LAIV included vaccine strains A/17/New York/15/5364(H1N1)pdm09, A/17/Hong Kong/2014/8296(H3N2), and B/60/Brisbane/08/83 (antigenic lineage B/Victoria). The distribution of serum antibody titers to NA and HA strain A/Hong Kong/4801/2014(H3N2) among 18 vaccinated patients is shown in Figure 4.

In Figure 4, it can be seen that the increase in the average titers of anti-HA antibodies to the vaccine strain A/17/Hong Kong/2014/8296 (H3N2) was significant, and the titers of antibodies to NA increased significantly (Figure 4). Among the 18 vaccinated persons, three (16%) responded to the vaccination with HI antibody seroconversions and nine (18%) with NI antibody seroconversions. Simultaneous conversion of antibodies to HA and NA occurred in only one case. Thus, antibodies to NA may serve as an additional criterion for assessing the immunogenicity of influenza vaccines. The GMTs among those who responded to vaccination were 56.6 for HI antibodies and 20.2 for NI antibodies, that is, they were not lower than after infection.

And finally, we wanted to compare the effect of age on the level of NI antibodies to N2 of the A/H2N2 and A/H3N2 viruses. To do this, we used archival samples of patients of different ages left over from routine laboratory tests conducted in 2013.

**Figure 5:**
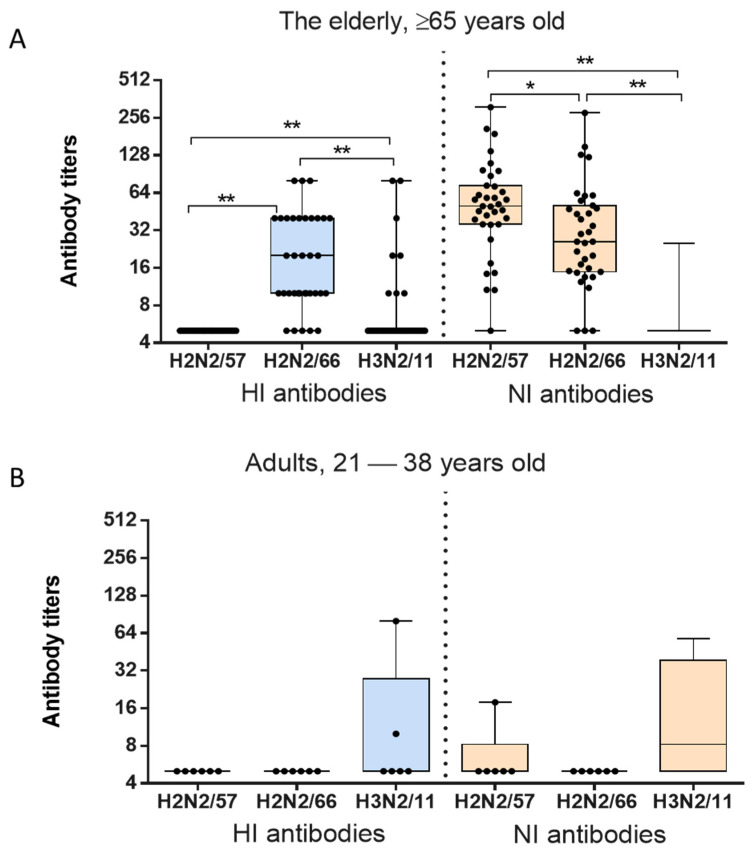
HI and NI antibody titers to A/Leningrad/134/17/1957(H2N2), A/California/1/1966(H2N2) and A/Victoria/361/2011(H3N2) in the sera of adults and elderly examined in 2013. (**A**). The elderly, ≥65 years old (*n* = 35). (**B**). Adults, 21–38 years old (*n* = 6). * *p* < 0.05, ** *p* < 0.01.

The levels of serum HI antibodies to A/Leningrad/134/17/1957(H2N2), A/California/1/1966(H2N2) and A/Victoria/361/2011(H3N2), shown in Figure 5A, differed significantly in elderly patients ≥65 years old (Friedman ANOVA: *p* < 0.0001). The highest main titer of HI antibodies was detected against the 1966 strain A/California/1/1966(H2N2), while the level of HI antibodies to A/Victoria/361/2011(H3N2) was four times lower (Wilcoxon Matched Pairs test: *p* = 0.0002). HI antibodies to A/Leningrad/134/17/57(H2N2) were not detected. The levels of anti-N2 antibodies to the influenza viruses of 1957, 1966 and 2011 were also statistically significantly different (Friedman ANOVA; *n* = 56, *p* < 0.00001) in the examined patients over 50 years of age (Figure 5A). The maximum mean titers of NI antibodies in this age group were detected against the strain A/Leningrad/134/17/57(H2N2). The level of serum antibodies to NA of A/California/1/1966(H2N2) was 1.6 times lower (Wilcoxon Matched Pairs test: *p* = 0.013). The study included six sera from adults aged 21–38 years which did not have HI antibodies to A/H2N2 viruses. Mainly antibodies to the HA and NA of seasonal influenza virus A/Victoria/361/2011(H3N2) were detected among these patients. Certain levels of NI antibodies to A/Leningrad/134/17/1957(H2N2) may be attributed to the cross-reactivity of antibodies to N2 of A/H3N2 viruses with which these patients may have previously been in contact.

The herd immunity to influenza viruses A/Leningrad/134/17/1957(H2N2), A/California/1/1966(H2N2) and A/Victoria/361/2011(H3N2) as assessed by antibody levels ≥1:40, are presented in Table 3. Despite the fact that there were no HI antibodies to A/Leningrad/134/17/1957(H2N2) influenza virus in the elderly, more than half of the examined patients had NI antibodies in titers ≥1:40. 34.3% of the examined elderly persons were seropositive to A/California/1/1966(H2N2) possessing HI antibody in titers ≥1:40 (Table 3). The same proportion of patients had antibody titers ≥1:40 to NA of A/California/1/1966(H2N2). In four out of 35 examined sera (11.4%), antibodies to HA and NA of A/California/1/1966(H2N2) were detected in titers ≥1:40 simultaneously. A medium-strength relationship was found between the titers of HI and NI antibodies (Spreaman’s correlation coefficient r^s^ = 0.525).

The percentage of seropositive elderly individuals to the A/Victoria/361/2011(H3N2) strain according HI test was only 8.6%, although none of examined elderly individuals had a titer of NI antibodies to seasonal influenza virus A/Victoria/361/2011(H3N2) ≥1:40.

## 3. Discussion

Although antibodies to influenza NA are neutralizing, antibodies to the second antigenic component–NA-can also provide protection, especially against viruses with novel HA variants. The most clear and substantiated results indicating the protective effect of NA antibodies were obtained during the flu pandemic in 1968 on a group of people with obviously absent or low levels of antibodies to the influenza virus A/Hong Kong/1/68(H3N2). Among the observed group of 274 people aged 20 to 45 years, the frequency of infection with the A/H3N2 influenza in November 1968–January 1969, detected by HI seroconversions, as well as severity, were inversely related to the level of antibodies to N2 [17]. It was then shown that NI antibodies with a titer ≥1:16 were associated with a 46.7% protective efficacy against infection. The relatively low level of herd immunity to NA strains A(H2N2) in the population explains the unhindered spread of the virus of the new subtype A(H3N2) in 1968; however, it could still result in moderate severity of the pandemic in question in comparison with the consequences of the expansion of the “Asian” flu in 1957 [18,19]. Despite an almost equal percentage of people infected in the pandemic seasons of 1968–1969 and 1969–1970, more than two-thirds of severe diseases and lethal outcomes accounted for the second epidemic wave caused by the pandemic strain with a significant drift of NA, but not HA. This involved the accumulation of 11 amino acid substitutions, three of which were localized in the NA antigenic sites [19].

Our study showed that minor changes in the structure of N2 can lead to changes in antigenic properties. We identified five amino acid substitutions located in the NA “head” differed between NA of viruses A/Hong Kong/4801/2014(H3N2) and A/Victoria/361/2011(H3N2). One such substitution at position 221 was described earlier. It was previously reported that an asparagine residue at position 221 changed to histidine when analyzing A/H2N2 and A/H3N2 influenza viruses isolated between 1957 and 1975 [20]. In our case, there was a substitution of the glutamic acid residue with aspartic acid. Viruses A/Hong Kong/4801/2014(H3N2) and A/Victoria/361/2011(H3N2) differed in antigenic properties, as was shown in the study of antibodies to these viruses in the sera of patients with influenza infection. Thus, in the same sera, NI antibody titers ≥1:20 to A/Victoria/361/2011(H3N2) and A/Hong Kong/4801/2014(H3N2) viruses occurred simultaneously in only 10% of cases. In our early studies, it was shown that changes in several amino acids affected the enzymatic activity and antigenic properties of N1 of A/California/07/2009(H1N1)pdm09 and A/South Africa/3626/13(H1N1)pdm09 [21]. Interestingly, the A/Victoria/361/2011(H3N2) virus has lysine (K) in position 258, and A/Hong Kong/4801/2014(H3N2) virus and other epidemic viruses have glutamic acid (E). Lysine at position 258 is also found in the A/Ann Arbor/6/60(H2N2) strain and has recently been described in the A/New York/PV190/2017(H3N2) influenza virus. This substitution is reported to reduce the sensitivity of escape mutants to H2-specific monoclonal antibodies [22]. It is possible that the presence of this substitution could affect the difference in the antigenic properties of the analyzed strains A/Victoria/361/2011(H3N2) and A/Hong Kong/4801/2014(H3N2).

The fact that groups of patients had a high level of antibodies to A/Hong Kong/4801/2014(H3N2) HA but almost no NI antibodies may indicate more frequent contacts with viruses of this subtype. The lack of formation of antibodies to N2 can be explained by the probable primary contact with the A/H1N1 viruses that caused the 1977 pandemic and continue to circulate until now. In the case of a change in the HA subtype, the group of those born in from 1977 to 2000 will be susceptible to influenza A/H3N2 viruses. Therefore, it could mean a need for priority vaccination of this age group with the appropriate vaccine strain.

It was shown that the intensity of serum antibody response after intranasal administration of trivalent LAIV was not lower compared to natural infection with the influenza A virus. It should have been taken into account that during immunization with LAIV, three vaccine viruses are introduced at once, to which an immune response is formed. Influenza infection, on the other hand, is supposed to be caused most often by one virus, but given the phenomenon of antigenic ‘sin’, antibodies may also form against previous variants with which the body has previously contacted. It was previously reported that the immune response to NA and HA differs between infection and vaccination. Thus, it has been shown that more NA-reactive than HA-reactive B cells are activated during H3N2 infection [23]. This is consistent with the data that NA is the most immunogenic influenza protein in terms of molar composition [24]. The presence of conserved epitopes in the NA molecule also provides for cross-reactivity with earlier isolates [25]. After vaccination, in contrast, the ratio of activated NA plasmablasts was very low. This was related only to the inactivated vaccine and was attributed to the inefficient presentation of immunogenic epitopes in the inactivated vaccine and the fact that the content of HA in the vaccine has not yet been standardized [23].

Seasonal strains of the A/H3N2 subtype, having significant antigenic differences in hemagglutinin with A/H2N2 strains that have left the circulation since 1968, represent the same NA subtype–N2. Influenza viruses of the A/H2N2 subtype are considered as potentially pandemic, since the majority of the population does not have immunity to them. This is also shown in our study in regard to HI antibodies. Thus, the presence of a group of people vaccinated with a seasonal trivalent live influenza vaccine (LAIV) of the composition A (H1N1) + A (H3N2) + B, or those who had a natural infection caused by A/H3N2 influenza, may be protected against the A/H2N2 pandemic strain at the initial stage until mass vaccination of the population is deployed. Previously, it was shown that the antibody titers against N2 and influenza B virus NA were moderately high compared to N1, which may be due to differences in stability between N1 and N2 [25]. The fact of immunological imprinting was also described, when middle-aged people had high titers of N2 antibodies to the A/H3N2 strain, which had long since left circulation, with which they could have come into contact in their youth. This phenomenon, also called primary antigenic ‘sin’, has been widely described for HA, but has not been sufficiently studied for NA [25].

Our data on high levels of antibodies to the A/California/1/1966(H2N2) virus in the elderly may suggest that a significant part of older people was in direct contact with the A/H2N2 virus isolated in 1966, in addition to similar viruses. The formation of a high level of NI antibodies to the A/California/1/1966(H2N2) in a large number of volunteers may also point to repeated contacts with other A/H2N2 viruses during the 1957 to 1968 circulation period. The cross-reaction of anti-N2 antibodies acquired during this period can explain the high percentage of individuals with a detectable titer of antibodies to the NA strain A/Leningrad/134/17/57(H2N2) in the absence of HI antibodies.

Data on the low antibody levels to the A/Victoria/361/2011(H3N2) in the elderly in 2013 may indicate a not very frequent contact with such viruses. A/H2N2 viruses isolated between 1957 and 1968 differ significantly from each other both antigenically and genetically [26]. The most significant deviations of antigenic characteristics from the properties of the 1957 pandemic pathogen occurred in strains isolated from 1965–1967. On the other hand, the antigenic differences accumulated in N2 over the past decades may lead to reduced cross-reactivity of N2 antibodies formed to influenza strains of 1957–1968 with the recent seasonal A/H3N2 influenza.

This study has potential limitations. We used ELLA to detect functional NA-inhibiting antibodies, while not taking into account non-NI antibodies that may potentially participate in Fc-mediated reactions.

## 4. Materials and Methods

### 4.1. Ethics Statement

In this retrospective study we examined blood sera from patients at the “IEM” Medical Research Center collected in 2018–2019 (*n* = 86), as well as paired sera from 18 healthy adults vaccinated with seasonal live influenza vaccine (LAIV) in the fall of 2017. Written informed consent was obtained from each subject. Also, in this study we used 15 pairs of sera from patients hospitalized during 2017–2018 flu season with PCR confirmed type A influenza. Additionally, we used 41 archive sera from healthy adults collected in 2013. All sera were left after routine lab tests. The study was approved by the Local Ethics Committee at Institute of Experimental Medicine (protocol dated 25 April 2019). After obtaining the approval of the local ethical committee, the sera were transferred to the researchers, none of whom had access to the personal data of the patients.

### 4.2. Viruses

To determine neuraminidase inhibiting (NI) antibodies in human sera, we used influenza A/H7N2 viruses that contained HA from A/horse/Prague/1/57(H7N7) and NA from A/Leningrad/134/17/1957(H2N2), A/California/1/1966(H2N2) or A/Victoria/361/2011(H3N2). One more virus was A/H6N2, which contained HA from A/herring gull/Sarma/51c/2006 (H6N1) and NA from A/Hong Kong/2014/8296(H3N2). To determine serum anti-HA antibodies, strains A/Leningrad/134/17/1957(H2N2), A/17/California/66/395(H2N2), A/17/Victoria/2011/89 (H3N2) and A/17/Hong Kong/2014/8296 (H3N2) obtained from the collection of the Institute of Experimental Medicine were used. All viruses were propagated in developing chicken eggs at 33 °C.

### 4.3. Determination of NA Inhibitory Antibodies

The levels of antibodies to NA of the A/H3N2 strains were evaluated using enzyme-linked lectin assay (ELLA) after determining the optimal conditions for the enzymatic reaction. For this, reassortant viruses A/H7N2 and A/H6N2 were purified by ultracentrifugation on a stepwise sucrose gradient. The working concentration of the virus was defined as 128 hemagglutinating units (HAU) in 50 μL. This dose provides a signal level when reading the results on a spectrophotometer in the range of optical density (OD) at 450 nm (OD450) from 0.45 to 0.70. The titer of serum NA inhibiting (NI) antibodies was calculated as the reciprocal of the dilution of the sample giving 50% inhibition of NA activity, i.e., a two-fold decrease in OD compared with the viral control when reading the results on the ELx800 spectrophotometer (BIO-TEK INSTRUMENTS, INC., Winooski, VT, USA).

### 4.4. Hemagglutination Inhibition Test (HI)

HI was performed according to the previously described method [27] in 96-well polymer plates for immunological reactions with a U-shaped bottom with a suspension of 0.75% human erythrocytes of the 0-group. The sera were pretreated with *Vibrio cholerae* neuraminidase extract (RDE, Denka Seiken, Japan). The reaction results were taken into account after erythrocyte sedimentation in control wells without virus. Serum anti-hemagglutination antibody titer was defined as the highest dilution that resulted in the inhibition of erythrocyte agglutination.

### 4.5. Assessment of Evolutionary Changes in Amino Acid Sequences and Molecular Phylogeny

Alignment of amino acid sequences taken from the database of influenza viruses of the National Center for Biotechnology Information (The Influenza Virus Resource at the National Center for Biotechnology Information) was performed using the UGENE program from UniPro (version 1.19.0) [28]. The designations of the strains used for the analysis of NA provided in Table 4. Evolutionary analyses were conducted in MEGA X [29].

The remote nearest neighbor method (neighbor joining) was used to reconstruct phylogenetic trees. The stability of the tree topology was assessed using bootstrap analysis based on the results of building and matching phylogenetic trees for 1000 new sets of amino acid sequences generated based on the original alignment.

### 4.6. Statistical Analysis of Results

The results were processed using the statistical package “Statistica” (version 6.0). The geometric mean titers (GMT), arithmetic mean (m) and standard deviation (σ) were used to describe the obtained data. For statistical analysis, antibody titers were expressed as log2 reciprocals of the final dilution. Checking the normality of the distribution was carried out using the Shapiro-Wilk test. If the assumptions about the normal distribution of the dependent variable within each group and the homogeneity of the variance were not met, Kruskal-Wallis rank analysis of variance was used. The comparison of dependent samples was carried out using the Wilcoxon test. To compare multiple independent groups, we used a Kruskal-Wallis analysis of variance (ANOVA) with subsequent multiple pairwise comparisons based on Kruskal-Wallis sums of ranks (Dunn test). For nominal data, Fisher’s exact two-tailed test was used. The presence of a statistical relationship between the variables was estimated using correlation analysis by the Spearman’s method. Criteria-tested null hypotheses were rejected at *p* < 0.05.

## 5. Conclusions

In this work, we analyzed the molecular and antigenic changes in NA subtype N2 with influenza infection and vaccination. The formation of NI antibodies in patients with acute influenza infection was studied. Using ELLA, antibodies to N2 were detected in patients of various ages. The lowest levels of antibodies to NA subtype N2 were noted in the group of volunteers born between 1977 and 2000, which may indicate the need for priority vaccination of this age group with the appropriate vaccine strain. In the group of patients vaccinated with LAIV, there was a significant increase in the average titers of NI antibodies to A/Hong Kong/4801/2014(H3N2). Thus, when evaluating the immunogenicity of an influenza vaccine, the increase in anti-NA antibodies should be taken into account.

## Figures and Tables

**Table 1:** Seroconversion to antigens of A/Victoria/361/2011(H3N2) and A/Hong Kong/4801/2014(H3N2) in paired sera of influenza patients.

Flu Season	Virus	HI Antibodies		NI Antibodies	
		% of ≥4-Fold Conversions	GMT among Seroconverters	% of ≥4-Fold Conversions	GMT among Seroconverters
**2017–2018** **(*n* = 15)**	A/Victoria/361/2011(H3N2)	33	31.74	13	57.25
	A/Hong Kong/4801/2014(H3N2)	27	45.79	33	66.71
2018–2019 (*n* = 20)	A/Hong Kong/4801/2014(H3N2)	15	63.5	30	27.6

**Table 2:** The level of herd immunity to antigens to HA and NA of A/Hong Kong/4801/2014(H3N2) in 2017–2018 flu season.

Years of Birth	HI Antibodies		NI Antibodies	
	GMT	% with Titers ≥1:40	GMT	% with Titers ≥1:20
1924–1956 (*n* = 29)	17.3	41.3	7.2	13.7
1957–1976 (*n* = 30)	13.5	20	5.9	10
1977–2000 (*n* = 27)	22.7	37	5.5	0

**Table 3:** The levels of ‘herd’ immunity to antigens to HA and NA of A/Leningrad/134/17/1957(H2N2), A/California/1/1966(H2N2) and A/Victoria/361/2011(H3N2) among elderly patients examined in 2013 (*n* = 35).

Influenza Virus	HI Antibodies (≥1:40)		NI Antibodies (≥1:40)	
	#	%	#	%
A/Leningrad/134/17/1957(H2N2)	0	0	20	57.1 ^1^
A/California/1/1966(H2N2)	12	34.3 ^2^	12	34.3
A/Victoria/361/2011(H3N2)	3	8.6	0	0

^1^ Proportion of patients with NI antibody levels ≥40 to A/Leningrad/134/17/1957(H2N2) significantly higher compared to A/California/1/1966(H2N2) (Fisher’s exact test, *p* = 0.045). ^2^ Proportion of patients with HI antibody levels ≥40 to A/California/1/1966(H2N2) significantly higher compared to A/Victoria/361/2011(H3N2) (Fisher’s exact test, *p* = 0.009).

**Table 4:** Influenza virus strains used for NA phylogenetic analysis.

	Strain NCBI Code	Strain Name	Subtype
1	L37330	A/Leningrad/134/17/1957	H2N2
2	AB296075	A/Singapore/1/1957	H2N2
3	AAO46219	A/Ann Arbor/6/1960	H2N2
4	AAO46235	A/California/1/1966	H2N2
5	KP098442	A/England/12/1962	H2N2
6	ACF54392	A/Georgia/1/1967	H2N2
7	KY321929	A/Hong Kong/01/1968	H3N2
8	AAA43419	A/Udorn/1972	H3N2
9	ABE12548	A/Port Chalmers/1/1973	H3N2
10	BAD16646	A/Texas/1/1977	H3N2
11	ABF21324	A/Bangkok/1/1979	H3N2
12	ADJ41808	A/Philippines/2/1982	H3N2
13	AAB06966	A/Mississippi/1/1985	H3N2
14	ABF21327	A/Leningrad/360/1986	H3N2
15	AAB06967	A/Shanghai/11/1987	H3N2
16	ABF21325	A/Beijing/353/1989	H3N2
17	ACF41859	A/Beijing/32/1992	H3N2
18	ACL12132	A/Shandong/9/1993	H3N2
19	AAB06998	A/Wuhan/359/1995	H3N2
20	CAC19707	A/Sydney/5/1997	H3N2
21	ABE73101	A/Moscow/10/1999	H3N2
22	AFH00640	A/California/7/2004	H3N2
23	ACF54579	A/Wisconsin/67/2005	H3N2
24	ACI26321	A/Brisbane/10/2007	H3N2
25	ACS71643	A/Perth/16/2009	H3N2
26	AIE52659	A/Ohio/02/2012	H3N2
27	ATV83197	A/Brisbane/1/2012	H3N2
28	AIE52623	A/Victoria/361/2011	H3N2
29	ATV84691	A/South Australia/55/2014	H3N2
30	EPI1868574	A/Hong Kong/4801/2014	H3N2
31	AQY20927	A/Texas/50/2017	H3N2
32	AQW37036	A/Colorado/06/2017	H3N2
33	AVG71505	A/Kansas/14/2017	H3N2

## Data Availability

Data is contained within article.
